# Reducing the Symptomatology of Panic Disorder: The Effects of a Yoga Program Alone and in Combination with Cognitive-Behavioral Therapy

**DOI:** 10.3389/fpsyt.2014.00177

**Published:** 2014-12-08

**Authors:** Camila Ferreira Vorkapic, Bernard Rangé

**Affiliations:** ^1^Laboratory of Neurophysiology, Department of Physiology, Federal University of Sergipe, São Cristóvão, Brazil; ^2^Department of Applied Psychology, Institute of Psychology, Federal University of Rio de Janeiro, Rio de Janeiro, Brazil

**Keywords:** anxiety, cognitive-behavioral therapy, contemplative practice, panic disorder, yoga

## Abstract

**Introduction:** Yoga is a holistic system of different mind–body practices that can be used to improve mental and physical health. It has been shown to reduce perceived stress and anxiety as well as improve mood and quality of life. Research documenting the therapeutic benefits of yoga has grown progressively for the past decades and now includes controlled trials on a variety of mental health conditions such as depression, anxiety, and panic disorder.

**Objectives:** The primary goal of this study was to investigate the effects of yoga in patients suffering from panic disorder. We aimed at observing the efficacy of yoga techniques on reducing the symptomatology of panic disorder (anxiety and agoraphobia), compared to a combined intervention of yoga and psychotherapy.

**Method:** Twenty subjects previously diagnosed with panic disorder were selected. Subjects were randomly assigned to both experimental groups: Group 1 (G1-Yoga: 10 subjects) attended yoga classes and Group 2 (G2-CBT + Yoga: 10 subjects) participated in a combined intervention of yoga practice followed by a cognitive-behavioral therapy (CBT) session. Both interventions occurred weekly for 100 min and lasted 2 months. Subjects were evaluated two times during the study: pre-test and post-test. Psychometric tools included the Beck Anxiety Inventory (BAI), Hamilton Anxiety Rating Scale (HAM-A), The Panic Beliefs Inventory (PBI), and Body Sensations Questionnaire (BSQ).

**Results:** Statistical analysis showed significant reductions in anxiety levels associated with panic disorder (G1: BAI – *p* = 0.035, HAM-A – *p* = 0.000; G2: BAI *– p* = 0.002, HAM-A – *p* = 0.000), panic-related beliefs (G1: PBI – *p* = 0.000; G2: PBI *– p* = 0.000) and panic-related body sensations (G1: BSQ – *p* = 0.000; G2: BSQ *– p* = 0.000) both in G1 and G2. However, the combination of yoga and CBT (G2) showed even further reductions in all observed parameters (mean values).

**Conclusion:** This study observed significant improvement in panic symptomatology following both the practice of yoga and the combination of yoga and psychotherapy. While contemplative techniques such as yoga promote a general change in dealing with private events, CBT teaches how to modify irrational beliefs and specific cognitive distortions. The results observed in G2 might indicate that the techniques complemented each other, increasing the intervention efficacy. These findings are in agreement with many investigations found in the literature which observed improvements in different mental health parameters after the practice of contemplative techniques alone or combined to psychotherapy. Future research joining psychological and physiological variables could help better elucidate the mechanisms through which mind-body practices work to improve mental health.

## Introduction

Anxiety disorders, which include panic disorder, are the most common class of mental disorders present in the general population. The estimated lifetime prevalence of any anxiety disorder is over 15%, while the 12-month prevalence is more than 10% (1). One study estimated the annual cost of anxiety disorders in the United States only to be approximately $42.3 billion in the 1990s (2, 3). Specifically, panic disorder, whose key element is an increase in anxiety level, is also a common mental disorder with significant clinical manifestations and socioeconomic impacts. Panic is characterized by the repeated occurrence of discrete panic attacks that features a variety of physical symptoms, such as rapid heartbeat, hyperventilation, perspiration, dizziness, dyspnea, trembling, and uncontrollable fear (fear of losing control and going crazy, fear of dying) (4, 5). Between attacks, patients might also develop persistent apprehension or anticipatory anxiety, regarding the possibility of another attack. In addition, about one half of these patients eventually develop agoraphobia (6).

A considerable amount of research has been carried out to quantify the magnitude of the short-term societal costs of anxiety disorders in general, in terms of healthcare expenditures, impaired functioning, and reduced longevity (7). The magnitude of the short-term societal costs of anxiety estimate in recent studies is surprising. Greenberg et al. (2) estimated that the annual total societal costs of active anxiety disorders in North America alone over the decade of the 1990s exceeded $42 billion. This estimate excludes the indirect costs of early-onset anxiety disorders through adverse life course outcomes (e.g., the documented effects of child–adolescent anxiety disorders in predicting low educational attainment and consequent long-term effects on lower income) and through increased risk of other disorders (e.g., anxiety disorders predicting the subsequent onset of cardiovascular disorder). Therefore, it has become urgent the need for effective low cost strategies that provide the right tools for patients to cope with anxiety themselves, in order to reduce the economic cost of mental disorder in society.

Current treatments for panic disorder include psychotherapeutic and pharmacological interventions, both supported by a great amount of empirical evidence (8–12). However, studies suggest that many individuals do not seek professional help, which indicates the need for reliable and appropriate self-help strategies (1). In addition, both patients and doctors agree on one point: it is not satisfactory to spend the entire life using drugs; and traditional psychotherapy can be costly when carried out for years, although some therapies such as the cognitive-behavioral therapy (CBT) are brief and effective (10–12).

Clinical trials have demonstrated that anxiolytic dugs have long-term limited efficacy (13), cause dependency, sleepiness (14) and sexual dysfunction (15, 16), and affect cognition and memory (14, 15). A national survey in the United States, carried out in the 1990’s examined the relationship between mental disorders and complementary or alternative medicine, such as acupuncture, meditation, and yoga (1). Sample (14.985 people) included patients with psychological disorders or those who have utilized the mental health services, as well as healthy individuals. Data analysis has shown that approximately two thirds of the sample has utilized some kind of complementary therapy with good results. Other studies in different countries have observed similar outcomes (17, 18). Nowadays, there is a consolidated therapeutic approach for treating mental disorder, but sometimes patients search for additional or complementary therapy for many reasons: adverse effects of drugs, lack of response to treatment, high cost of psychotherapy, or just preference for an integrative approach (19). One of these complementary therapies includes *mind-body* interventions, such as meditation and yoga.

Yoga is an ancient mind/body practice originated in India millennia ago. Although, according to traditional scriptures, its ultimate goal is the achievement of a unified state of consciousness and self-realization, yoga might be used to improve overall health and well-being (20). Yoga practice involves different techniques such as physical postures (*asana*), controlled breathing (*pranayama*), deep relaxation (*yoganidra*), and meditation (20). These techniques seem to have a specific influence on one’s mental state (21, 22). Research on the psychophysiological benefits of yoga and meditation has demonstrated significant improvements in emotional self-regulation with consequent reductions in depression, stress, and anxiety levels (21, 23–27); as well improvements in mood (28), quality of life and well-being (29). According to Balasubramaniam et al. (29), the efficacy of yoga as a therapeutic intervention has led to the popular implementation of yoga as a primary or adjunctive therapy in the treatment of mental disorders.

Research documenting the therapeutic benefits of yoga has grown progressively for the past decades and now includes controlled trials and the presence of active control groups on a variety of mental health conditions such as depression, anxiety, and panic disorder (30–32). A bibliometric analysis of published research studies on yoga as a therapeutic intervention has revealed almost 50 published studies up to 2003 evaluating yoga for mental health conditions, all of which reported positive benefits (33). A recent review of yoga for neuropsychiatric disorders concluded that there is emerging evidence to support the benefits of yoga for depression, sleep disorders, and as an augmentation therapy (29).

Different studies that have used yoga as a main therapeutic approach demonstrated the efficacy of yoga techniques in reducing stress (8, 25, 34, 35), anxiety (8, 25, 34–42), depression (25, 40), and stress-related symptoms such as hypertension and insomnia (8). In addition to not being a pharmacological intervention, yoga-based therapy has no adverse effects (if so, minimum), it is a self-help strategy that can be practice by the individual once properly learned and it is well accepted internationally (43).

Based on the last decades of evidence regarding the effectiveness of contemplative techniques (yoga, meditation) for mental disorders, mental health professionals have begun to draw their attention to these eastern practices. In the last years, psychologists elaborated a promising therapeutic approach that integrates meditation and CBT (44). It is said that eastern (both Hindu and Buddhist) interpretation for suffering is intimately related to cognition-based deductions regarding the “I,” and that some ideas, such as the consequences of actions, are clearly reflected on CBT techniques that emphasize positive behavior as part of the therapy (45).

The Mindfulness-Based Cognitive Therapy (44) is the first attempt to professionally combine the efficacy of the eastern and western approaches in the treatment of mental disorder. It is an integration of CBT practices and perspectives with meditation, in which individuals have the opportunity to observe their thoughts, emotions, and physical sensations in the absence of “catastrophic consequences” (44).

According to Douglass (46), mental health professionals recognize that contemplative techniques such as yoga may be a beneficial therapeutic supplement to psychotherapy, but they are not entirely informed about why or how these practices are effective. Thus, mental health professionals may benefit from trustworthy controlled studies and investigations in order to properly incorporate this learning into their practice with patients and offer a more comprehensive and desired mind–body therapeutic treatment.

Although, there are several studies demonstrating the legitimacy of this approach (8, 25, 34–42), no investigation, to date, has observed the efficacy of the combination of yoga and CBT in a complete manner (the complete yoga practice, not some elements incorporated by CBT); as well as the comparison to yoga alone in treating mental disorder.

Therefore, the primary goal of this study was to investigate the effects of yoga in patients suffering from panic disorder. This investigation aimed at observing the efficacy of yoga techniques in reducing the symptomatology of panic disorder compared to a combined intervention of yoga and psychotherapy.

## Materials and Methods

### Subjects

Twenty subjects between 18 and 60 years, both male and female, attendees of the Psychology Institute Ambulatory of the Federal University of Rio de Janeiro were selected for the study. Subjects previously diagnosed with panic disorder (DSM IV), with or without agoraphobia, were properly screened to participate in the study using the Mini International Neuropsychiatric Interview (MINI 6.0) (47). Individuals suffering from severe pulmonary disease, heart condition or high blood pressure were excluded from the investigation. Subjects with depression (comorbidity) or making use of antidepressant or anxiolytic drugs were included. All participants answered initial forms and questionnaires that included demographic data, substance use, general health, and informed consent, according to the Psychiatry Institute Ethics Committee (CEP/UFRJ) protocols. Table 1 shows demographic data.

**Table 1:** **Demographic characteristics of G1 and G2 subjects**.

	G1 (Yoga)	G2 (Yoga + CBT)
Percent female	99.0	99.0
Age (years)	42.3 (7.54)	40.9 (9.19)
Education (years)	13.1 (2.00)	14.2 (2.03)
**Marital status**		
Single	3 (35%)	3 (35%)
Married	6 (60%)	6 (60%)
Divorced/widowed	1 (5%)	1 (5%)

*(), SD*.

### Experimental protocol

Distribution to group assignment occurred after baseline measure completion (psychological evaluation). Participants were allocated to two treatment conditions: Group 1 (G1-Yoga) attended yoga classes and Group 2 (G2-CBT + Yoga) participated in a combined intervention of yoga practice and CBT session. A separate examiner, not the main researchers, coordinated the group distribution (and all statistical analysis was performed by an expert from another state). But since the main examiners were directly responsible for the interventions, the composition of the groups was known to the investigators at some point.

G1 attended yoga sessions two times a week and each class lasted 50 min. G2 attended yoga practices and CBT sessions twice a week for 100 min (yoga practice once a week = 50 min; CBT session once a week = 50 min). Hatha Yoga techniques included body postures (*asana*), breathing techniques (*pranayama*), relaxation (*yoganidra*), and meditation (*mindfulness*).

Clinical research rooms had furniture and medical instruments and were not carpeted. So, in order to facilitate a better pleasant atmosphere, yoga sessions were carried out at a partner yoga studio nearby the Psychology Institute. Yoga sessions followed a small yoga treatment manual. All contemplative practices were administered by the first author, which is a certified yoga teacher.

Both interventions lasted two consecutive months. All subjects were patients from the Psychology Institute Ambulatory who sought these health units due to their severe condition, so assigning them to a passive control group such as waiting list (no intervention) could worsen their mental state. Therefore, it was chosen to compare two active groups, with different interventions.

### Psychological assessment

Subjects were evaluated two times during the study: pre-test and post-test. Psychometric tools included: The Beck Anxiety Inventory (BAI) (48); Hamilton Anxiety Rating Scale (HAM-A) (49); The Panic Beliefs Inventory (PBI) (50); and Body Sensations Questionnaire (BSQ) (50).

Additionally, to examine the impact and acceptance of yoga, subjects answered a small feedback questionnaire [adapted from Ref. (51)], which included items such as reasons for participating in the study, suggestions, motivations and teacher evaluation.

### Statistical analysis

The statistical software Statistical Package for the Social Sciences (SPSS) – version 17.0 was used to analyze research data. Histograms were visually inspected in order to observe distribution features of the continuous variables. Normality distribution was also analyzed by asymmetry and kurtosis, as well as by a Shapiro–Wilk test. Due to the small number of the sample, a non-parametric Wilcoxon statistics was utilized when pertinent. For categorical variables such as demographic data, absolute (N) and relative (%) frequencies were calculated. Data were also analyzed in terms of central tendency (mean, median) and variability (standard deviation, variance, and mean). Afterward, the associations between experimental times (pre- and post-test) and groups (G1 and G2) were tested using a model analysis of variance (ANOVA) on participants’ questionnaires scores.

## Results

The hypothesis that both interventions would reduce the symptomatology of panic disorder over time was confirmed by the results. Significant time effects were found across both groups on all variables, indicating that regardless of group membership, scores improved from time 1 (pre-test) to time 2 (post-test). Group-by-time interaction effects were found on scores for anxiety [BAI: *F*(1, 35) = 9.29 *p* = < 0.001; HAM-A; *F*(2, 30) = 10.28, *p* = < 0.001], panic beliefs [*F*(2, 35) = 10.92, *p* = < 0.001], and body sensations [*F*(1, 31) = 9.90 *p* = < 0.001].

There were no main effects of group and no significant interactions on these outcome measures. Essentially, participants improved over time in both groups. The pattern of results on all measures was highly similar, with improvements over time being greater in the G2 than in G1 (mean values). Table 2 presents the means, standard deviations, and *p-*values of all psychological parameters’ scores (except BAI) for both groups at each assessment point.

**Table 2:** **Means and SD of the psychological parameters’ scores for both treatment groups at each assessment point**.

Outcome measure	Time	Yoga			Yoga + CBT		
		Mean	SD	*p*	Mean	SD	*p*
HAMA-A	Pre-test	31.45	±1.7	*p* < 0.001	29.06	±1.4	*p* < 0.001
	Post-test	16.05	±2.7		12.55	±1.7	
PBI	Pre-test	44	±2.2	*p* < 0.001	42.7	±1.4	*p* < 0.001
	Post-test	26.95	±2.1		20.03	±2.2	
BSQ	Pre-test	49.8	±1.3	*p* < 0.001	51.20	±1.3	*p* < 0.001
	Post-test	30.01	±1.6		24.90	±2.4	

**Figure 1:**
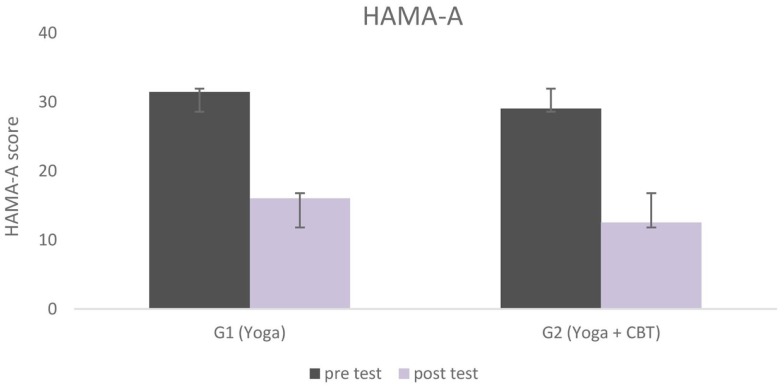
**Hamilton anxiety rating scores for both groups, from baseline to end program**.

**Figure 2:**
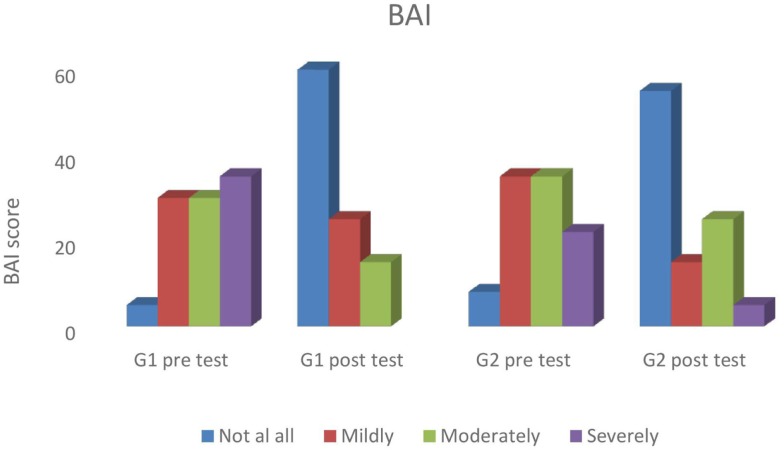
**Percentage values for all levels of anxiety (BAI) for both groups across experimental time**.

Figure 1 shows statistically significant changes in anxiety (HAM-A) from baseline to end-program (pre- and post-test) in both groups. Participants’ scores in both groups also improved according to the BAI; with subjects going from clinical to subclinical categories (*not at all, mildly, moderately, and severely*) in the course of the interventions. Categorical analysis showed statistical difference at the significance level of 5% in the BAI in both groups; after both the Yoga (G1, *p* = 0.035) and the Yoga + CBT interventions (G2, *p* = 0.002). According to Beck and Steer (52), a total score of 0–7 indicates minimal level of anxiety (*not at all*), 8–15 indicates mild anxiety (*mildly*), 16–25 indicates moderate anxiety (*moderately*), and 26–63 indicates severe anxiety (*severely*) (*cut off* points). Percentage values of all levels of anxiety (BAI) for both groups across experimental time are presented in Figure 2.

**Figure 3:**
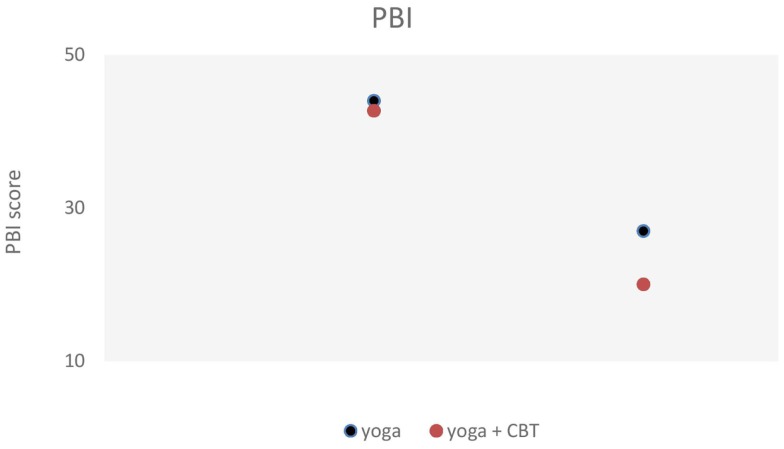
**Shows statistically significant changes in panic-related beliefs (panic belief inventory) from baseline to end-program (pre- and post-test), for both experimental groups**.

Results of the statistical analysis also showed time effects across conditions (both groups) for panic-related beliefs. Subjects scored significantly lower at the PBI after both interventions, although G2 (Yoga + CBT) showed greater improvements (mean values) (Figure 3).

**Figure 4:**
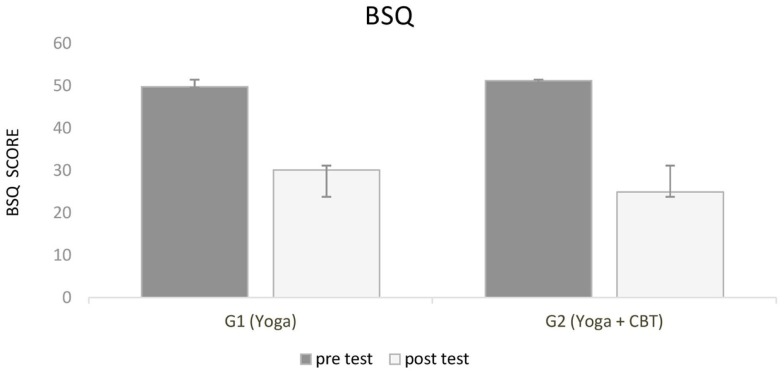
**Shows the results for panic-related body sensations (body sensations questionnaire)**. Scores improved significantly from pre- to post-test in both groups.

Panic-related body sensations evaluated by the BSQ significantly improved from pre to post-test in both groups as well. Although no significant differences were observed between groups, G2 (Yoga + CBT) presented lower score at BSQ (Figure 4).

To summarize, the ANOVA showed time effects across conditions (G1 and G2) on all panic-related variables (anxiety, panic beliefs, and body sensations), indicating that observed symptoms improved in both groups over time. The differences between the groups were not significant, although greater improvements over time were observed in G2 compared to G1 (mean values).

## Discussion

Anxiety and anxiety-related symptoms have a huge short-term societal cost. Although current treatment for anxiety (and mental disorder in general) includes the consolidated drug-psychotherapy approach, its consequences include: adverse effects of drugs, lack of response to treatment, high cost of psychotherapy, or and pharmacological interventions (30). Hence, it has become increasingly urgent the need for effective low cost strategies, which provide the right tools for patients to cope with anxiety themselves, changing the individual’s entire lifestyle and reducing, therefore, the economic cost of this mental disorder in society.

One of these strategies embraces integrative approaches such as mind–body interventions, like meditation and yoga. Yoga practice involves different techniques (physical postures, controlled breathing, deep relaxation, and meditation) that seem to have a specific influence on one’s mental state (21, 42). Research on the overall benefits of yoga practice has revealed improvements in emotional self-regulation with consequent reductions in depression, stress and anxiety levels (25, 26); as well improvements in mood (53), quality of life and well-being (29). Balasubramaniam et al. (29) have reviewed the evidence for yoga for mental disorders and have concluded that there is emerging evidence to support the benefits of this practice.

After decades of evidence regarding the effectiveness of contemplative techniques for mental disorders, health professionals are now beginning to utilize them as complementary or main therapy. In the last years, a promising therapeutic approach that integrates meditation and CBT was formulated by psychologists and has been showing positive results (44, 54–60). Although there are many studies demonstrating the validity of this approach (44, 55–62), the current investigation is the first to observed the efficacy of the combination of yoga and CBT in a complete manner (as described earlier); as well as the comparison to yoga alone in treating mental disorder.

Therefore, the primary goal of this study was to investigate the effects of yoga in patients suffering from panic disorder. This investigation aimed at observing the efficacy of yoga techniques on reducing the symptomatology of panic disorder compared to a combined intervention of yoga and psychotherapy.

Data analysis demonstrated improvements in panic-related symptoms such as anxiety, body sensations, and mental constructs for both groups, indicating the effectiveness of yoga and the combination of yoga with CBT as treatment interventions. There were no main effects of group and no significant interactions on these outcome measures, which means that participants improved over time in both groups. Although the pattern of results on all measures was highly similar, improvements over time were greater in G2 than in G1 (mean values). Such improvements in mood, anxiety, quality of life, and mental health in general after the practice of contemplative techniques were also observed in many studies and are well established in the literature (8, 22, 25, 34, 37, 39, 40, 42, 45, 55).

According to neurophysiological studies, the constant activation of the sympathetic nervous system (SNS) and the hypothalamic–pituitary–adrenal axis (HPA) produces chronic stress, anxiety, and depression, affecting the integrity of the entire brain (63). Alternatively, contemplative practices such as yoga, seem to have the capacity to reduce this activation and thus might be valuable in treating mental disorders (33). The relaxation response observed after the practice of yoga is the result of reduced arousal level and SNS activity and the activation of antagonistic neuromuscular and limbic systems (64). In addition, short and long-term yoga practice is associated with reduced cortisol levels, catecholamine secretion basal metabolic rate and oxygen consumption (33).

The relaxation response, through which yoga seems to relieve anxiety and stress, might be related to other systems, such as the regulation of breathing (65, 66). Yoga techniques teach how to voluntarily control breathing, influencing unconscious mechanisms and consequently modulating the interaction between the sympathetic and parasympathetic systems, as well as the HPA axis (65).

Yoga practice could also be involved in a particular class of behavior that increases vagal influence in the body (67). According to the Porges’ polyvagal theory, physiological states support different classes of behavior. For example, a physiological state characterized by vagal withdraw would support the fight or flight behavior. On the other hand, a state differentiated by the intensification of vagal influence would induce spontaneous social engagement. When the environment is perceived as safe, motor parasympathetic fibers (vagal motor pathway) increase their influence on the body, slowing down heartbeat, inhibiting the fight or flight response, reducing the SNS activity and cortical release (67).

The practice of *pranayama* (breathing techniques) can, in addition, desensitize CO2 chemoreceptors in brainstem so that they respond lesser to the regular CO2 increase during exhaling, until the point that the individual is able to exhale slowly and still reduce the heartbeat. Such practice alters the breathing patterns in such a deep manner, that further reductions in heartbeat are also seen when the individual is not practicing (65). Therefore, breathing adjustment is possibly one of the reasons for the efficacy of yoga to increase relaxation and positive feelings and reduce anxiety (65).

Although the mechanisms through which yoga act are still not clear, one thing seem to be certain: evidence show that yoga practitioners are less predisposed to developing anxiety disorders and respond better to negative emotions when they appear (68). However, yoga should be considered as complete intervention (with all its techniques). If the different aspects are administered separately, such reductionist approach could result in loss of efficacy.

The combination of contemplative techniques with psychotherapy, such as CBT, as a therapeutic intervention protocol has also been investigated in the last decades. In fact, different studies have demonstrated the efficacy of this combination in treating mental disorder (61, 62). A recent study observed the effects of eight weeks of MCBT (mindfulness-based CBT) in patients suffering from generalized anxiety disorder. Results show significant reductions in anxiety levels from the beginning until the end of the intervention (61). Weiss et al. (62) compared the effectiveness of psychotherapy (group 1) and psychotherapy combined with MBSR (mindfulness-based stress reduction program) (group 2). Results show that both groups reduced depression, anxiety, and stress levels, but participants in group 2 were able to formulate their goals clearer, better adhered to the program and finished psychotherapy earlier.

Similarly, the present study observed slightly better results (mean values) in panic symptomatology when integrating yoga to CBT. The efficacy of this combination might be due to the fact that CBT and contemplative techniques, such as yoga, share similar concepts, although exposed differently. While meditative practices promote a general change in the way an individual deal with private events, CBT teaches how to modify irrational beliefs and identify cognitive distortions, as well as fix them (69, 70). However, according to Segal et al. (44), there are different aspects of the contemplative practices that were already implicitly present in CBT. During cognitive remodeling, the individual learns that what he thinks about himself is not always the correct representation of reality, that his thoughts are far from being trustworthy and objective information and that an attitude change influences emotional reactions (44). Yoga (which includes meditation) promotes a form o negative thought awareness, in which the qualities of acceptance, decentralization, and detachment cultivate the inner capacity to reflect and influence cognitive experiences. The constant practice of this view might help to regulate emotions through an increase in cognitive flexibility (55).

Therefore, it is possible that the interventions complemented each other, increasing their efficacy to some extent and reducing panic-related symptoms. These findings are in agreement with other investigations found in the literature, which observed improvements in different mental health parameters after the practice of contemplative techniques alone or combined to some type of psychotherapy (25, 27, 28, 34, 40, 42, 44, 55–60).

Future research joining psychological and physiological variables could help better elucidate the mechanisms through which *mind-body* practices work to improve mental health.

## Conflict of Interest Statement

The authors declare that the research was conducted in the absence of any commercial or financial relationships that could be construed as a potential conflict of interest.
